# Cortisol values during the standard-dose cosyntropin stimulation test: Personal experience with Elecsys cortisol II assay

**DOI:** 10.3389/fendo.2022.978238

**Published:** 2022-08-18

**Authors:** Hasan Husni, Mohammed S. Abusamaan, Roshan Dinparastisaleh, Lori Sokoll, Roberto Salvatori, Amir H. Hamrahian

**Affiliations:** ^1^ Division of Endocrinology, Diabetes, and Metabolism, Johns Hopkins University School of Medicine, Baltimore, MD, United States; ^2^ Department of Pathology, Johns Hopkins University School of Medicine, Baltimore, MD, United States

**Keywords:** cosyntropin stimulation test, adrenal insufficiency, new cortisol assay, elecsys cortisol II assay, secondary adrenal insufficiency, ACTH stimulation test

## Abstract

**Purpose:**

There has been debate regarding the appropriate cortisol cutoff during the cosyntropin stimulation test (CST) when newer cortisol assays are used. We aimed to evaluate the proper cortisol values during the standard dose CST in patients with normal hypothalamic-pituitary-adrenal (HPA) axis when the Elecsys^®^ Cortisol II assay from Roche Diagnostics is used.

**Methods:**

We retrospectively reviewed the medical records of patients evaluated for possible adrenal insufficiency using the standard-dose (250 mcg) CST from January 2018 to December 2020 and eventually judged to have a normal HPA axis. All the CSTs were done in the outpatient setting. Evaluation by an endocrinologist, restrictive exclusion criteria including prior glucocorticoid and opioid use, and lack of glucocorticoid treatment for at least 6 months after the CST was used to define normal HPA axis. The results are reported in the median (range).

**Results:**

We identified 63 patients who met the inclusion criteria and were considered to have a normal HPA axis. The median age was 54.7 (27.6-89.1) years; 32 (51%) were female, and 27 (43%) were white. The duration of follow-up after the CST without any glucocorticoid replacement was 13.9 (6.3-43.9) months. Cortisol levels were 21.7 (15.7-29.1) µg/dl and 24.4 (17.9-35.8) µg/dl at 30- and 60-minutes after cosyntropin administration, respectively. The lowest cortisol levels at 30 and 60 minutes for patients with either normal TSH or gonadal axis (n=47) or in whom both axes were normal (n=18) were similar to the ones of the entire cohort.

**Conclusion:**

Our study supports using a lower than previously recommended cortisol cutoff value at 30 minutes after Cosyntropin using the Roche Elecsys^®^ Cortisol II assay. The lowest cortisol levels in our cohort were 15.7 and 17.9 µg/dL at 30 and 60 minutes after the CST, respectively. Therefore, it is essential to consider the time of cortisol draw after cosyntropin administration.

## Introduction

Adrenal insufficiency (AI) is associated with significant morbidity and can be life-threatening. The biochemical diagnosis of primary AI is often clear and can be established by demonstrating a low morning cortisol level and an elevated plasma ACTH. However, secondary AI can be more challenging to diagnose, as the cortisol values can fall into the indeterminate zone, accompanied by low or apparently “normal” ACTH levels. The Insulin tolerance test (ITT) is considered the gold standard test to diagnose AI. The metyrapone test has shown a good correlation with the ITT. However, both tests have been largely replaced by the cosyntropin stimulation test (CST) in clinical practice due to its ease and safety (1, 2).

Serum cortisol can be measured by Liquid chromatograph-mass spectrometry (LC-MS/MS) or by immunoassay. LC-MS/MS is considered to be more specific. However, immunoassays are simpler to perform and more readily accessible. Different from the first generation assay, the Elecsys^®^ Cortisol II assay from Roche Diagnostic (Cortisol II) uses monoclonal antibodies and is standardized to an isotope dilution–gas chromatography/mass spectrometry (ID-GC/MS) traceable reference material. Traditionally, a peak cortisol level of > 18-20 µg/dl during the standard-dose CST has been accepted to rule out adrenal insufficiency in non-critically ill patients using standard immunoassays (3, 4). Recently however, there has been debate about the cortisol cutoff values during the CST, as the results may vary based on the assay used.

Recent studies suggested using lower cortisol cutoffs if the newer assays are used (5–12). This has been attributed to the higher specificity of the newer cortisol assays than the older standard immunoassays, which can cross-react with other glucocorticoids such as 11-deoxycortisol, cortisone, and corticosterone (13). Cortisol cutoffs as low as 12.7 µg/dL have been suggested as the pass criteria during the standard dose CST (11). Some of the suggested cortisol cutoffs, especially at 60 minutes post-CST, are not in line with our experience using the Cortisol II assay. A lack of strict exclusion criteria, and combining low dose- and standard dose-cosyntropin tests may have been among potential confounding factors in some of the studies suggesting the lower cortisol cutoffs.

In this retrospective study, using strict exclusion criteria we aim to evaluate the appropriate cortisol values during the standard-dose CST in patients with normal hypothalamic-pituitary-adrenal (HPA) axis during real-life clinical practice using the Cortisol II assay from Roche Diagnostics.

## Methods

### Patient population and design

This was a retrospective chart review study using electronic medical records. The study included adult patients (age 18 years and older) who had the CST performed in the outpatient setting of the Johns Hopkins health system from January 2018 through December 2020. They had ICD-10 codes of pituitary gland disorders in their problem list or diagnoses at the time of data extraction. Serum cortisol was tested in the Johns Hopkins Medical Laboratories with the Elecsys Cortisol II electrochemiluminescence immunoassay (Roche Diagnostics). The cortisol levels were measured at 30 and/or 60 minutes after IM or IV administration of 250 µg cosyntropin. The intra- and inter-assay coefficients of variation for cortisol II assay on the e801 analyzer are 1.1-5.5% and 1.7-7.3%, respectively.

All patients were evaluated by an endocrinologist at Johns Hopkins University and were eventually judged to have a normal HPA axis. A normal HPA axis was defined based on evaluation by an endocrinologist, restrictive exclusion criteria including prior glucocorticoid and opioid use, and a lack of need for glucocorticoid replacement for at least 6 months on follow-up visits. We used the electronic health record (EHR) EpicCare^®^ database, the Chesapeake Regional Information System for our Patients (CRISP), and “Care Everywhere” system to assure that none of the patients received any glucocorticoids during their follow-up. “CRISP” is the designated Health Information Exchange in Maryland and the District of Columbia designated to allow instant sharing of health information among doctors’ offices, hospitals, labs, radiology centers, and other healthcare organizations. “Care Everywhere” is used within the Epic Hyperspace patient record, to exchange electronic health records with outside organizations. It provides access, to a patient’s medical records from other organizations. In addition, we analyzed the cortisol levels at 30 and 60 minutes after cosyntropin injection in subsets of in patients with either normal TSH or gonadal axis or in whom both axes were normal.

**Figure 1:**
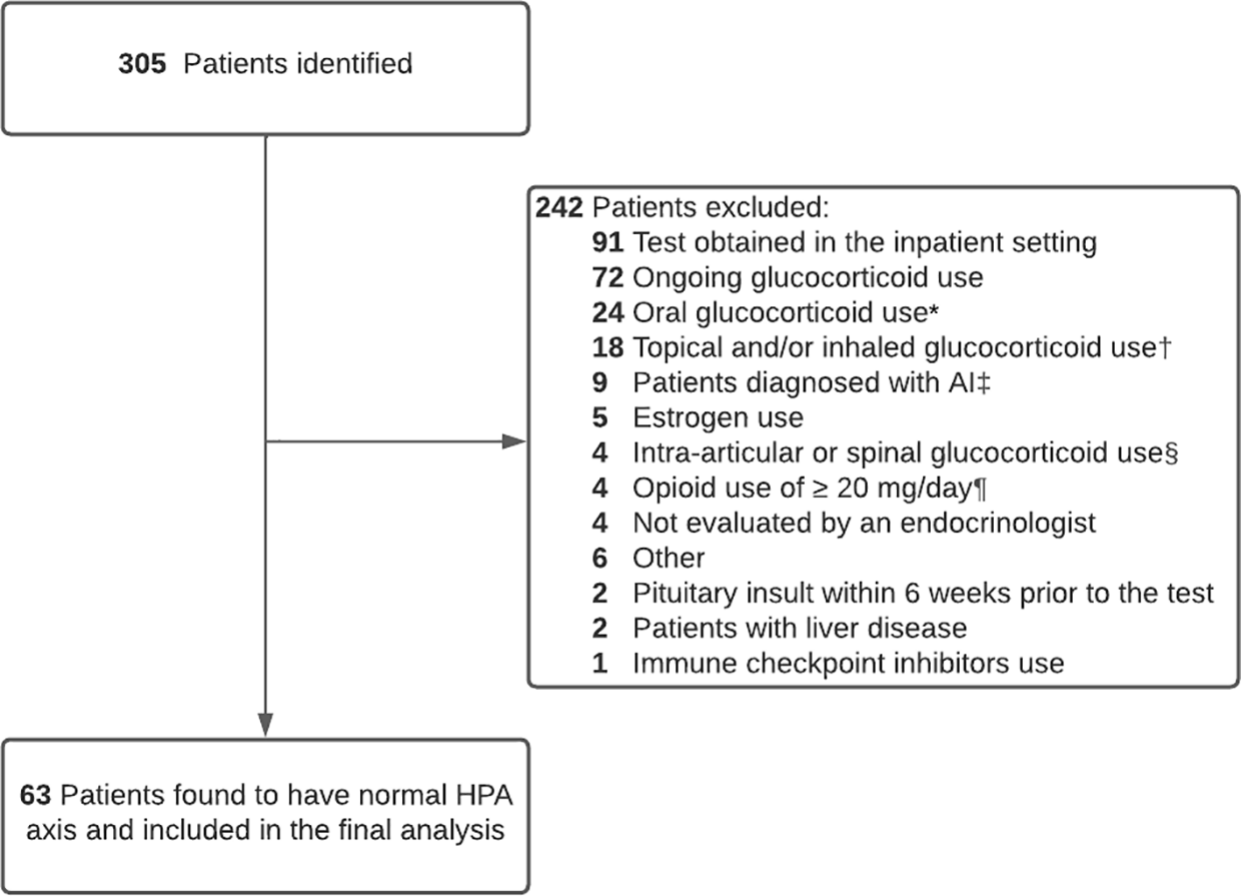
Study Flow Chart of 63 patients who were included in the final analysis of the study. * Oral glucocorticoid use for more than a week within 6 months prior to the CST† Topical or inhaled glucocorticoid use for more than a week within 3 months prior to the CST‡ Patients who were considered to have AI on initial evaluation by the treating endocrinologist. § Intra-articular or spinal glucocorticoid injection use within 3 months prior to the CST, or if the patient received more than 2 injections within 12 months prior to the CST¶ Opioid use at doses of morphine milligram equivalent (MME) ≥ 20 mg/day within 3 months prior to the CST.

The CST was carried out for evaluation of the HPA axis. Patients were excluded from the analysis if they had one or more of the following: a) any ongoing glucocorticoid use regardless of route, b) topical or inhaled glucocorticoid use for more than a week within 3 months before the CST, c) oral glucocorticoid use for more than a week within 6 months before the CST, d) intra-articular or spinal glucocorticoid injection within 3 months before undergoing the CST, or if they received more than 2 injections within 12 months before the CST, e) serum albumin < 2.5 g/dL, f) patients with liver disease such as hepatitis, g) pituitary insult such as pituitary surgery within 6 weeks before performing the CST, h) oral estrogen use within 3 months before performing the CST, and pregnant women, i) opioid use at doses of morphine milligram equivalent (MME) ≥ 20 mg/day within 3 months before the CST (14); j) previous use of immune checkpoint inhibitors within 2 years of the CST. The study flowchart is shown in **Figure 1**.

### Data sources and measures

The patients’ medical record numbers and demographics were extracted from our electronic health record. The study was approved by the Johns Hopkins Institutional Review Board. Data were de-identified after extraction. Given the retrospective nature, no informed consent was deemed necessary.

We collected the data regarding the CST date and time, cortisol concentrations at baseline and then 30-, and 60- minutes post-cosyntropin administration. In addition, the status of the gonadotropin and thyrotropin axes (when available) were also collected. A normal thyrotropin axis was defined based on having a normal free T4 and TSH levels. A normal gonadotropin axis was defined as having normal menstrual cycles in premenopausal women, appropriate follicular stimulating hormone (FSH) in postmenopausal women (>25.8 mIU/mL), and normal testosterone in men (>300 ng/dl). The variables were included if they were collected within a maximum period of one year from the stimulation test. In most cases, these evaluations were done simultaneously or close to the date of the CST. We also reviewed the follow-up visit notes, including the date of follow-up, to detect patients who were started on glucocorticoids.

### Statistical analysis

Descriptive statistics were used to summarize the patient characteristics for the study population. Number and percentage were reported for categorical variables. For continuous measures, the normality of data was assessed using histograms and tests of skewness and kurtosis. The results for the continuous measurements are reported in the median (range). Statistical analyses were performed using Stata Statistical Software: Release 15 (College Station, TX: StataCorp LP).

## Results

Among 305 patients who underwent the CST, 63 met the inclusion and exclusion criteria and were found to have a normal HPA axis (**Figure 1**). The median (range) age was 54.7 (27.6-89.1) years; 32 (51%) were females, and 27 (43%) were white. **Table 1** shows the characteristics of patients included in the study. The cortisol values at 30- and 60-minutes post-cosyntropin administration were available for 43 patients (68.2%) and 61 patients (96.8%), respectively.

**Table 1:** Patients characteristics and cortisol levels at baseline, and then 30- and 60 minutes after the cosyntropin stimulation tests.

Factor	(N = 63)
Sex: Female, no. (%)	32 (51)
Race, no. (%)	
White	27 (43)
Black	20 (32)
Other	16 (25)
Age, median (range)	54.7 (27.6-89.1)
Cortisol 0 minute, µg/dL, median (range)	9.9 (4.1-18.6)
Cortisol 30 minute, µg/dL, median (range)	21.7 (15.7-29.1)
Cortisol 30 minute, µg/dL, median (2.5%-97.5%)	21.7 (15.7-27.4)
Cortisol 60 minute, µg/dL, median (range)	24.4 (17.9-35.8)
Cortisol 60 minute, µg/dL, median (2.5%-97.5%)	24.4 (18.4-33.2)

39 patients had cortisol 0-minute value.

43 patients had cortisol 30-minute value.

61 patients had cortisol 60-minute value.

**Figure 2:**
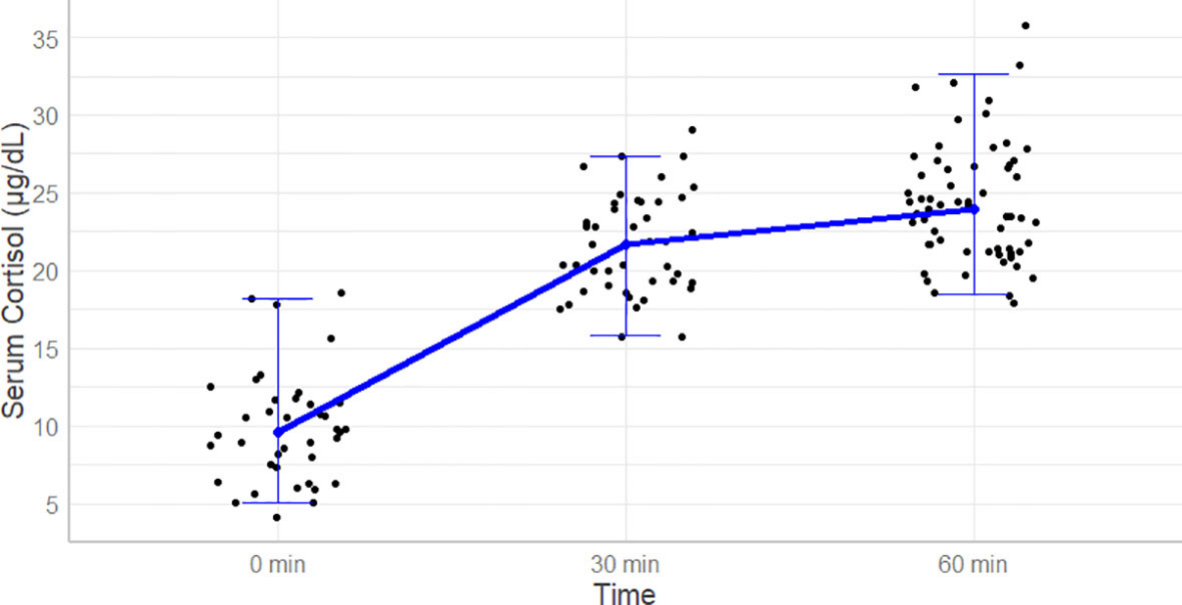
Cortisol levels at baseline and then 30 and 60 minutes after the Standard Dose Cosyntropin Test. The solid line connects the median values. The whiskers represent 95% of the data.

The cortisol concentrations were 21.7 (15.7-29.1) µg/dl and 24.4 (17.9-35.8) µg/dl at 30- and 60-minutes after cosyntropin administration, respectively (**Table 1** and **Figure 2**). Among the 63 patients, 60 (95%) and 53 (84%) patients had data for the evaluation of the thyrotropin and gonadotropin axes, respectively. The lowest cortisol concentrations at 30 and 60 minutes after cosyntropin in patients with either normal TSH or gonadal axis (n=47) or in whom both axes were normal (n=18) were similar to the ones of the entire cohort. After excluding the lower 2.5% of the data, the cortisol cutoff values at 30- and 60-minutes after the CST were 15.7 and 18.4 µg/dL, respectively (**Table 1**). The duration of follow-up after the CST was 13.9 (6.3-43.9) months. None of the patients were started on glucocorticoid therapy.

There was no significant difference between the 30- and 60 minutes cortisol values in patients younger and older than 60 years of age, in those with BMI more or less than 30, the time of performing the test (morning vs. afternoon), and with the sex of the patients (data not shown).

Using a traditional cortisol pass criterion of 18 µg/dL, 5/63 (7.9%) patients would have failed the CST at 30 minutes. Only one patient had a cortisol level <18 µg/dL (17.9) at 60 minutes. In all except three patients, 60-minute cortisol values were higher than 30-minute values.

## Discussion

In this study, we used very restrictive exclusion criteria including any significant previous glucocorticoid or opioid exposure prior to the CST. All the patients in this study who were eventually judged to have normal HPA axis had cortisol levels higher than 15 and 18 µg/dL at 30- and 60-minutes post standard CST, respectively, using the Elecsys^®^ Cortisol II assay.

Assessment of the HPA axis is an important task often required by endocrinologists. An early morning serum cortisol concentration of < 2-3 µg/dL is highly suggestive of AI (6, 15–18). On the other hand, a morning cortisol level of > 10-12 µg/dL in non-critically ill subjects makes adrenal insufficiency very unlikely, provided cortisol-binding globulin is not elevated (15, 19–22). Patients with cortisol values between these cutoffs require further evaluation by the CST to assess the HPA axis. 

Several studies have shown a 20-30% decrease in cortisol concentrations using the Cortisol II assay compared with older assays based on polyclonal antibodies. Accordingly, a lower pass criteria of 12.7-17.5 µg/dL has been suggested during the CST (5, 7, 9–12). There are several important factors that should be considered when reviewing the recent literature that suggests using lower cortisol cutoffs. These include the timing of the cortisol blood draw during the CST (30 min vs. 60 min), the use of low (1 µg) vs. standard (250 µg) dose CST, the inclusion of hospitalized patients, and a lack of restrictive exclusion criteria such as prior glucocorticoid and opioid exposure.

In some of the studies, the time of the cortisol blood draw in its interpretation during the CST is not analyzed (7, 9, 11). As shown in the literature and confirmed in our study, the 60-minute cortisol values are almost always higher than 30-minute cortisol levels after the standard (250 µg) Cosyntropin dose. Cortisol levels as high as 22 µg/dL have been used as the pass criteria at 60 minutes using older cortisol immunoassays (2–4, 23). Accordingly, it is essential to differentiate the pass criteria at 30- and 60-minutes post-cosyntropin, primarily since some clinicians may only obtain a single value after cosyntropin at either 30 or 60 minutes.

Kline et al. performed 82 dynamic adrenal stimulation tests, including low- and standard-dose CSTs, glucagon stimulation tests, and insulin tolerance tests (7). All samples were run simultaneously using the older Cortisol I and the newer Cortisol II assays, and a subset of samples were also analyzed using LC-MS/MS. A normal response was defined as a cortisol level > 18 µg/dL during any part of the stimulation test. Using an ROC curve for the Cortisol II assay, they found out that using a cortisol cutoff of 12.69 µg/dL would be associated with a sensitivity of 91% and specificity of 97%. Several studies argue against using the same cortisol cutoff during the low dose CST and the Glucagon Stimulation Test (2, 7, 24–27). Additionally, the Kline study included neonates and pediatric patients, and no data on the timing of the blood sample during the CST was provided (7).

Javorsky et al. enrolled 110 patients undergoing standard-dose CST using the Elecsys Cortisol I, Elecsys Cortisol II, Beckman Coulter Access, and LC-MS/MS assays (6). The authors suggested using a cortisol cutoff of 14.6 µg/dL for the 30-minutes post-cosyntropin value. The authors did not propose a cortisol pass criteria at 60 minutes, but they noted that the 60-minute cortisol value was higher than the 30-minute value. The study included samples from both ambulatory and inpatient settings. The value of the CST in the inpatient setting, especially in critically ill patients, is not fully established. The authors acknowledged a lack of follow-up of the patients as one of the limitations of their study. Our suggested cortisol pass criteria at 30 minutes is slightly higher than the level suggested by these authors.

Ueland et al. performed standard-dose CST in 121 healthy individuals using LC-MS/MS (10). Samples from 54 healthy subjects were also analyzed using the Roche Cortisol II assay. The median cortisol values were higher at all time points for immunoassays than for LC-MS/MS. The authors recommended values of 14.93 µg/dL for the 30-minute and 17.58 µg/dL for the 60-minute post-cosyntropin administration using LC-MS/MS. These cortisol cutoffs are similar to our findings.

Peechakara et al. compared the 1 μg IV low-dose, 25 μg IM medium-dose, and 250 μg IM standard-dose cosyntropin tests in a small prospective study to evaluate patients for secondary adrenal insufficiency. The insulin tolerance test was used as the gold standard test. Cortisol levels were measured using LC-MS. Using the ROC curve, the best cortisol cutoff at 30- and 60-min post standard CST were 16.1 and 19.5 µg/dL, respectively, which is in line with our finding that similar cortisol cutoffs cannot be used at 30 and 60 minutes time-point during the CST (2).

In a more recent publication by Zha et al. the authors compared serum cortisol levels in 50 patients evaluated for adrenal insufficiency who underwent CST. The serum cortisol levels were measured by Abbott Architect, Roche Elecsys II, and LC-MS/MS using polyclonal antibody-based Siemens assay as the reference method (12). Cortisol measurements with the Abbott monoclonal assay were similar to those from LC-MS/MS and Roche Elecsys II, but significantly lower than Siemens. The authors recommended using a cortisol cutoff level for the Architect of 13.2 µg/dL at 30 minutes and 14.6 µg/dL at 60 minutes based on a “normal” peak cortisol level of 18 µg/dL for the Siemens Centaur assay. As it was discussed before, cortisol levels as high as 22 µg/dL have been used as the pass criteria at 60 minutes using older cortisol immunoassays (2–4, 23). This may have been one of the reasons for suggesting a lower cortisol cutoff at 60 minutes by Zha study (12). The strength of our study lies in the extensive strict exclusion criteria to eliminate factors that could interfere with the cortisol levels such as recent glucocorticoid exposure, opioid use, and conditions that may be associated with low cortisol-binding globulin.

There are several limitations to our study, including its retrospective design and lack of gold standard comparisons such as the Insulin Tolerance Test or Metayrapone Test. The endocrinologists who evaluated the patients likely used the result of the CST as part of their assessment of the integrity of the HPA axis. Some of the endocrinologists only requested cortisol levels at baseline and 60 minutes during the CST. Accordingly, only 68% of the patients had cortisol values available at 30 minutes. The additional pituitary hormonal evaluation was not available in the whole cohort due to the retrospective nature of the study. Larger prospective studies and the inclusion of gold standard reference tests would provide us with more definite pass criteria during the CST using newer cortisol assays.

One of the limitations of this study is the lack of glucocorticoid use during the follow-up as one of the criteria for the presence of a normal HPA axis. None of the patients received any glucocorticoids for a median follow-up of about 14 months (minimum of 6 months). We acknowledge however that an even longer duration of follow-up and record of going through significant life stress (such as major surgical procedures) could be even stronger evidence for the presence of a normal HPA axis. We used additional criteria including the assessment of other pituitary axes for the integrity of the pituitary function. We conducted a sub-analysis in 47 patients who had normal HPG or HPT axes, and 18 patients who had normal HPG and HPT and found similar cortisol cutoffs for the CST.

Our study did not include a cohort of healthy subjects. However, the patients represent the real-life scenario in the outpatient setting to evaluate adrenal insufficiency. In addition, the previous studies in healthy subjects have suggested similar cortisol pass criteria to ours (10).

## Conclusion

Using restrictive exclusion criteria, our study agrees with the recent literature that a lower cortisol cutoff value should be used at 30 min after Cosyntropin using the Roche Diagnostics Elecsys^®^ Cortisol II assay. However, our result does not support using a cortisol value of 15 µg/dL as pass criteria at 60 minutes. Therefore, it is essential to consider the time of cortisol draw after CST for appropriate evaluation of the HPA axis. Future larger prospective studies can provide us with a better refinement of the cortisol cutoffs using the new assays.

## Data availability statement

The raw data supporting the conclusions of this article will be made available by the authors, without undue reservation.

## Ethics statement

The studies involving human participants were reviewed and approved by Johns Hopkins Institutional Review Board. Written informed consent for participation was not required for this study in accordance with the national legislation and the institutional requirements.

## Author contributions

AH and HH contributed to the study conception and design. HH wrote the primary draft of the manuscript. HH, MA, and RD performed data collection, and analysis. RS, MA, RD, and LS contributed to the manuscript. AH supervised the preparation of the manuscript. All authors contributed to the article and approved the submitted version.

## Conflict of interest

The authors declare that the research was conducted in the absence of any commercial or financial relationships that could be construed as a potential conflict of interest.

## Publisher’s note

All claims expressed in this article are solely those of the authors and do not necessarily represent those of their affiliated organizations, or those of the publisher, the editors and the reviewers. Any product that may be evaluated in this article, or claim that may be made by its manufacturer, is not guaranteed or endorsed by the publisher.
